# Deaths from Anæsthetics

**Published:** 1898-02-12

**Authors:** 


### DEATHS FROM ANÆSTHETICS.

(*Continued from page* 328.)

We now conclude the long list of deaths from anæsthetics, or occurring during anæsthesia, which took place in the course of the past year. Long as is the list, however, it does not entirely cover the whole mortality in England from this cause, for when we began to collect these cases we gave our attention to chloroform alone. But it soon became obvious that the reputation of ether for safety was not entirely borne out by facts, and it will be observed that in the second half of the year a not inconsiderable number of deaths during the administration of ether are included.

There is, however, a marked difference between the deaths under ether and those under chloroform. Most of the ether cases died after the anæsthetic had been administered for some time, and in some cases death clearly took place in consequence of the severity of the operation. Only in one case, in fact, does it appear that death took place under ether before the operation had been commenced. Very different is the history of the chloroform cases. No single fact is more striking than the frequency with which death took place at once, apparently as the direct result of the special action of the drug. It seems impossible to avoid the conclusion that in these cases the dose was too great, at any rate too large for the individual in question.

Then comes the great question: Is this over-dose the result of certain patients being peculiarly susceptible to the influence of chloroform, so that a very small dose becomes with them an over-dose; or is it the result of faulty methods of administration, which allow the vapour to be administered in too concentrated a form? The experience of the laboratory, as well as the fact that in some of these cases of sudden and unexpected death the patients had previously taken chloroform without any difficulty, all points to the conclusion that it is the accidental absorption of too concentrated a vapour that is the cause of death in accidents from chloroform, and we cannot but feel that whenever chloroform is administered some means should be taken to ensure exactitude of dose and evenness of strength of vapour, things which seem impossible of attainment by the ordinary method of pouring the drug on a cloth or a piece of lint and leaving it to evaporate as it likes.

The *Durham County Advertiser*, October 8th, reports the death of a youth, aged 15 years, at Durham, while under chloroform. The operation (for phymosis) had just been completed.

The *Morning Advertiser*, October 10th, reports an inquest on a girl, aged 12 years, who died at Hornsey Road, after an operation for a tumour. Gas and then ether were administered. The patient did not recover consciousness, and died from syncope, which was attributed to the shock of the operation.

The *Morning Leader*, October 17th, reports an inquest on a woman, aged 37, who died at the Temperance Hotel while under ether. The patient suddenly expired just as the operation was commenced. Post-mortem examination showed the existence of disease of the heart and lungs.

The *Sussex Daily News*, November 1st, reports an inquest on a woman, age 46, who died at the Worthing Infirmary while chloroform was being administered for an operation on a tumour in the abdomen. The operation had not been commenced, and she had not got fairly under the chloroform when she died.

The *British Medical Journal*, November 6th, reports the death of a man aged 62, at the Salop Infirmary, Shrewsbury, while under chloroform for the removal of cancerous glands in the neck.

The *People*, November 14th, reports the death of a child, aged four years, at the Middlesex Hospital, while under chloroform for operation for contracted fingers. The operation was nearly completed when death took place.

The *British Medical Journal*, November 27th, reports the death at St. Thomas's Hospital of a boy, aged 15 years, while under chloroform. The operation (circumcision) was almost completed when death took place.

The *Liverpool Daily Courier*, November 27th, reports the death at Rock Ferry of a woman under chloroform, administered for an operation two days after a premature confinement.

The *Medical Times and Hospital Gazette*, December 11th, reports the death of a boy, aged eight years, in the Stanley Hospital, Liverpool, after an operation for which chloroform was administered.

The *Birmingham Daily Post*, December 11th, reports the death of a man at the Corbett Hospital, Stourbridge, after an operation under ether. He was under observation for ten minutes after the operation, and was then removed to the ward. About half an hour after the ether had been administered the porter reported his death.

The *Barrow Herald*, December 11th, reports the death at Barrow of a child, aged nine years, while under chloroform for removal of adenoids.

The *Sussex Daily News*, December 14th, reports the death of a girl, aged 16, at the Sussex County Hospital, while under ether. She had been under ether about ten minutes when death occurred.

The *Daily News*, December 17th, reports the death of a woman, aged 44 years, at the Hospital for Women, Euston Road, while under ether, which had been preceded by nitrous oxide, for an operation for cancer. She had been under the anæsthetic about 40 minutes when she died. The patient was in a very bad state on her admission.

The *Birmingham Daily Post*, December 20th, reports the death of a child, aged five years, at the General Hospital, while under chloroform for the removal of tonsils and adenoids. The A.C.E. mixture was first given, and then chloroform.

The *Worcester Chronicle*, December 26th, reports the death of a child, aged eight years, at the Kidderminster Infirmary, while under chloroform. Death took place almost directly after the child was under the influence of the chloroform.

The *Birmingham Daily Gazette*, January 4th, 1898, reports the death of a man, aged 40 years, at the Queen's Hospital, on December 31st, whilst under chloroform for an operation on the kidney. The operation was not commenced.

